# Evaluating the treatment of patients with paroxysmal nocturnal hemoglobinuria in Brazil: disparities in access and treatment persistence

**DOI:** 10.1016/j.htct.2026.106459

**Published:** 2026-04-27

**Authors:** Rodolfo Delfini Cançado, Paula de Melo Campos, Diego Kashiura, Felipe Thies, Erica Ferreira, Ana Beatriz Machado de Almeida, Lays Paulino Leonel, Lucas Vieira Cortez, Nina Melo, Fabio Fedozzi, Alessandro Bigoni

**Affiliations:** a Hematology-Oncology Department, Faculdade de Ciências Médicas da Santa Casa de São Paulo - FCMSCSP, São Paulo, SP, Brazil; b Hematology and Hemotherapy Center, Universidade Estadual de Campinas – Unicamp, Campinas, SP, Brazil; c Novartis, São Paulo, SP, Brazil; d Real World Evidence, IQVIA Brazil, São Paulo, SP, Brazil; e Brazilian Lymphoma and Leukemia Association, São Paulo, SP, Brazil

**Keywords:** Paroxysmal Nocturnal hemoglobinuria, Eculizumab, Treatment persistence, Geographic access, Quality of life

## Abstract

**Background:**

Paroxysmal nocturnal hemoglobinuria is a rare, life-threatening disease that significantly affects quality of life. Despite therapeutic advances, challenges like treatment burden and limited care access persist. This study aimed to evaluate the geographic distribution of paroxysmal nocturnal hemoglobinuria patients in Brazil and explore how travel distance to treatment centers may influence treatment persistence.

**Methods:**

A mixed-methods study was conducted combining quantitative analysis of administrative claims data from the Brazilian Unified Health System and qualitative insights from patient focus groups. The quantitative component included patients with at least one ICD-10 D59.5 code between January 2010 and December 2023. The qualitative component involved a focus group of adult paroxysmal nocturnal hemoglobinuria patients registered with the Brazilian Lymphoma and Leukemia Association (ABRALE). Descriptive statistics were used to summarize demographic, geographic, and treatment-related data.

**Results:**

A total of 769 patients were identified, with 42.4% receiving eculizumab. The mean time to treatment initiation was 172.9 days. Spatial analysis revealed substantial regional variations, with eculizumab-treated patients traveling an average of 87.5 km for care. Persistence analysis showed that 21.2% and 61.0% of patients were non-persistent based using two different methodological approaches. Focus group participants reported limited disease awareness, long travel distances, and extended waiting times, all of which negatively impacted their daily lives and treatment adherence.

**Conclusion:**

This study highlights geographic disparities in access to paroxysmal nocturnal hemoglobinuria treatment in Brazil and suggests that the travel burden may contribute to treatment non-persistence. These findings underscore the need for more accessible and patient-centered therapeutic options to promote equitable healthcare delivery.

## Introduction

Paroxysmal nocturnal hemoglobinuria (PNH) is a rare disease, with a global prevalence ranging from 1-5 cases per million individuals [1]. PNH is typically diagnosed in adults aged 30 to 40 years. It can occur as a primary condition or concurrently with other bone marrow disorders, most commonly aplastic anemia and myelodysplastic syndromes [2].

Some authors suggest that PNH is underestimated because of low diagnosis rates in individuals with limited symptomatology [3]. The main clinical findings include hemolysis, thrombosis, and bone marrow disorders such as aplastic anemia or myelodysplastic syndromes [4]. Some patients classified as having PNH may have small clones that do not require treatment. However, patients commonly experience chronic fatigue, abdominal pain, and other symptoms that significantly impact their quality of life [4,5].

Currently, several complement inhibitors are approved, each targeting distinct components of the complement cascade. These include terminal pathway inhibitors directed at component 5 (C5) - such as ravulizumab, eculizumab, and crovalimab - as well as proximal or alternative pathway inhibitors targeting C3 (pegcetacoplan), factor B (iptacopan), and factor D (danicopan). These agents also vary in their routes of administration and dosing schedules [6].

Eculizumab was the first approved treatment for patients with PNH [7]. In Brazil, it was approved in 2017 [7], recommended for reimbursement in the Public Healthcare System (SUS) in 2018, and implemented in 2022 [8]. According to PNH Clinical Protocols and Treatment Guidelines (PCDT), the eculizumab regimen includes an initial and maintenance phases. During the initial phase, eculizumab is administered as an intravenous infusion once a week for the first four weeks. This is followed by the maintenance phase with infusions every two weeks [9].

Ravulizumab, a new C5 inhibitor, was approved by the Brazilian National Agency for Sanitary Surveillance in 2019, incorporated into the public system in 2024, and awaits the publication of the new PCDT to make it available for prescription in public services [10].

Brazil faces disparities in access to care [11]. In a country of continental proportions, the distance to care is likely a significant contributor to health inequalities [12]. However, there are no data describing the impact of demographic characteristics on the treatment of PNH patients in the Brazilian public health system.

## Objective

This study aimed to assess geographic features and the regional distribution of PNH patients in Brazil. Additionally, it analyzed the distance PNH patients traveled to receive eculizumab treatment in infusion centers and analyzed this travel burden with treatment persistence.

## Methods

The study used a mixed-methods approach, incorporating both quantitative and qualitative methodologies. It consisted of two parts: a retrospective database analysis and a focus group study.

### Study design and database

The quantitative component was a retrospective analysis of claims databases from the Department of Informatics of the Brazilian Unified Health System (DATASUS) with data extracted from the Hospital Admissions Information System (HAIS) and Outpatient Procedures Information System (OPIS).

The databases of HAIS and OPIS are not linked by a unique identifier; therefore, a probabilistic record linkage was used in this analysis. This linkage used different combinations of patient information from both databases as has been used previously; these included the date of birth, sex, race, city, and postal code [13].

### Study population

Patients were identified by the presence of at least one claim with the ICD-10 code D59.5 (PNH) of the International Classification of Diseases, 10th edition (ICD-10) in either the HAIS or OPIS from January 1st, 2010, to December 31st, 2023. The index date was defined as the date of the first ICD code for PNH.

Patients were classified as not treated with eculizumab if they did not have a High Complexity Procedures Authorization record for the drug during the study period (2010–2023), despite having at least one ICD-10 code D59.5. This absence was interpreted as a true lack of treatment within the SUS system, rather than as missing data.

### Treatment duration

Time from the index date to treatment initiation was calculated in days. For patients treated with eculizumab, initiation was defined as the date of first eculizumab record. For patients not treated with eculizumab, it was defined as the first PNH-related procedure, that is, any medical procedures associated with PNH management and recorded under the ICD-10 code D59.5, including transfusions and diagnostic procedures. Treatment duration was calculated from the first to the last record of eculizumab infusion or PNH-related procedure, respectively.

### Distance analysis

**Fig. 1:**
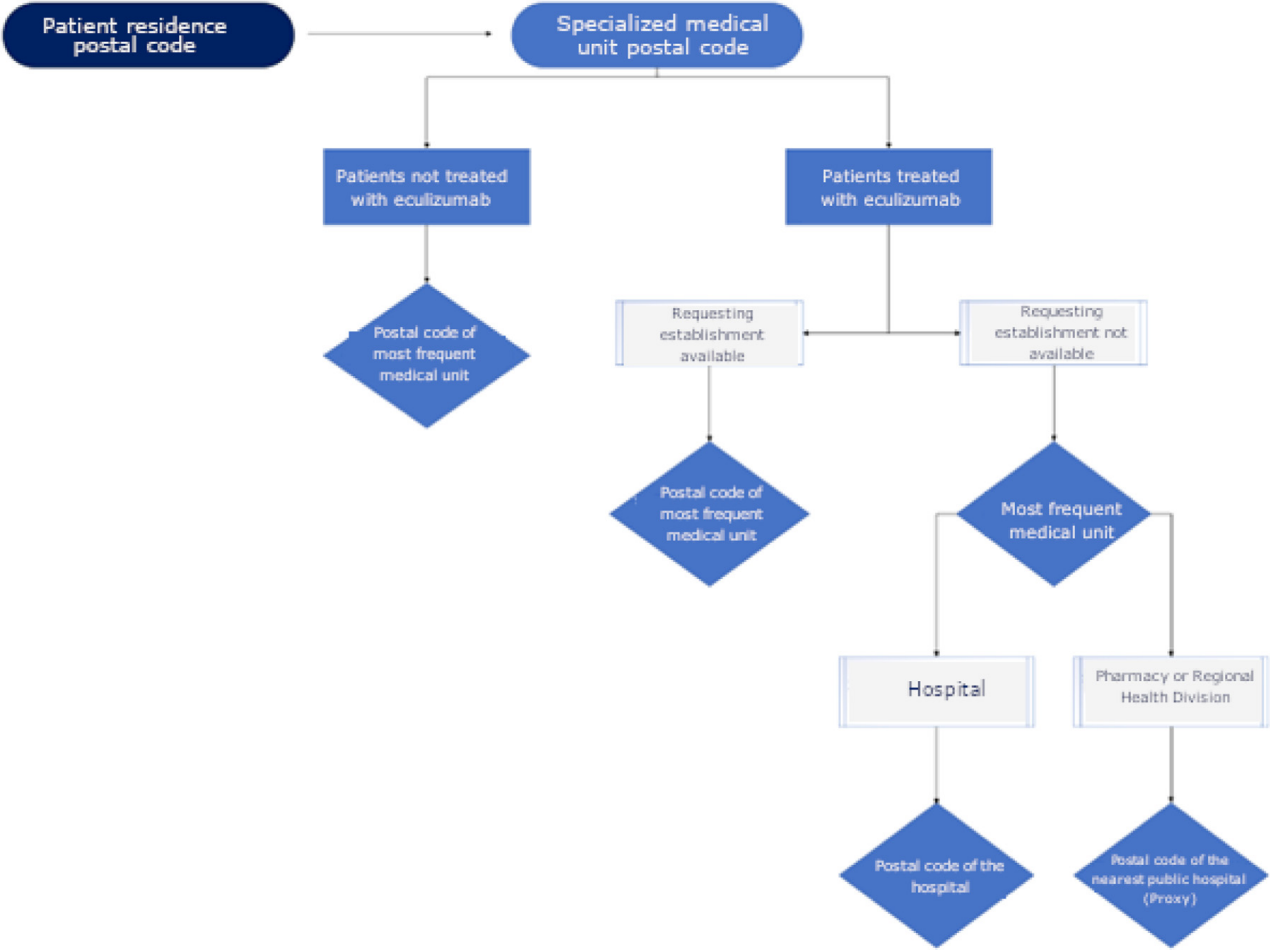
Analysis of the distance between the patient's residence and the treatment unit. The flowchart shows the criteria used for measuring the distance according to postal code of the patient's residence and the specialized unit, obtained from databases of the Department of Informatics of the Brazilian Unified Health System (DATASUS). **Note:** *If the patient presented discrepant postal code information, the most frequent one was considered.

The distance between the patient’s residence using the postal code and specialized medical units for treatment was calculated by the Google Maps application program interface (Figure 1). Google Maps uses satellite and aerial imagery to calculate the distance between two points. If postal code information was unavailable, the distance was not measured.

For patients not treated with eculizumab, the distance was calculated between the patient’s residence and the most frequently specialized medical unit for treatment.

For patients treated with eculizumab, the distance was calculated from the patient’s residence to the requesting establishment for eculizumab treatment. If this information was unavailable, the most frequently recorded specialized medical unit was used. When this unit was a hospital, its postal code was used directly. If it was a pharmacy or a regional health division, facilities that do not provide infusion services, the postal code of the nearest public hospital (general or specialized in hemotherapy) was used as a proxy. This approach ensured that distance calculations reflected access to clinically meaningful treatment settings.

### Eculizumab persistence analysis

The persistence analysis was measured for patients who received eculizumab treatment based on two approaches:

### Approach A: monthly consistency

Each patient was followed from the first to the last eculizumab record or the end of the study period, whichever came first. Patients were classified as ‘persistent’ if they had at least one eculizumab record in each calendar month during the follow-up. If there was any month without a recorded infusion, the patient was classified as ‘non-persistent’. This approach reflects monthly treatment continuity rather than average treatment intervals.

### Approach B: infusion interval consistency

This method calculated the average interval between the first and last eculizumab records by dividing the total time span by the number of infusions. Patients were classified as 'persistent' if the mean interval between infusions was less than or equal to 15 days, consistent with the recommended maintenance dosing schedule. If the average interval exceeded 15 days, they were considered 'non-persistent'.

In both approaches, the date of eculizumab records were extracted from the High Complexity Procedures Authorization records.

### Data analysis

All eligible records were analyzed; therefore, no sample size calculation was performed. This study was based on the entire population registered in DATASUS. Continuous variables were described using central tendency and dispersion measures, and categorical variables were expressed as absolute numbers and frequencies. Missing data were reported as such, and no extra data imputation was performed. Statistical analysis was performed using Python version 3.11.0.

Follow-up time was calculated from the index date (first ICD-10 D59.5 claim) to the last available record in the database. Treatment duration was calculated independently, based on the first and last eculizumab infusion records, which may precede or extend beyond the index date.

### Focus group

The focus group was part of an independent initiative conducted by ABRALE. The inclusion criteria for participants in the focus group were: 1) being registered in ABRALE's contact system; 2) having a diagnosis of PNH; 3) agreeing to participate by signing the informed consent form; and 4) being over 18 years old. Patients were excluded if: 1) they had passed away, or 2) they had incomplete registration data.

The focus group involved six patients with PNH from different states in Brazil. This meeting was held virtually using the Microsoft Teams platform. Patients were invited via phone contact, with the informed consent form being sent to them. The group was conducted by a researcher from ABRALE following a structured script prepared in Microsoft Word. The questions aimed to explore patients' perceptions and experiences regarding the impact of the disease, the process of accessing treatment, their knowledge about the condition, and the challenges faced in their treatment. The responses were analyzed through discourse analysis and tabulated in Microsoft Excel.

### Ethical considerations

The quantitative part of this study was conducted based on the administrative claims databases from DATASUS, which is publicly made available by the Brazilian Ministry of Health. The focus group conducted by ABRALE was submitted and approved by the ethics committee of ABRALE (CAAE: 80532324.0.0000.0071). This study was conducted in compliance with local laws and regulations.

## Results

**Fig. 2:**
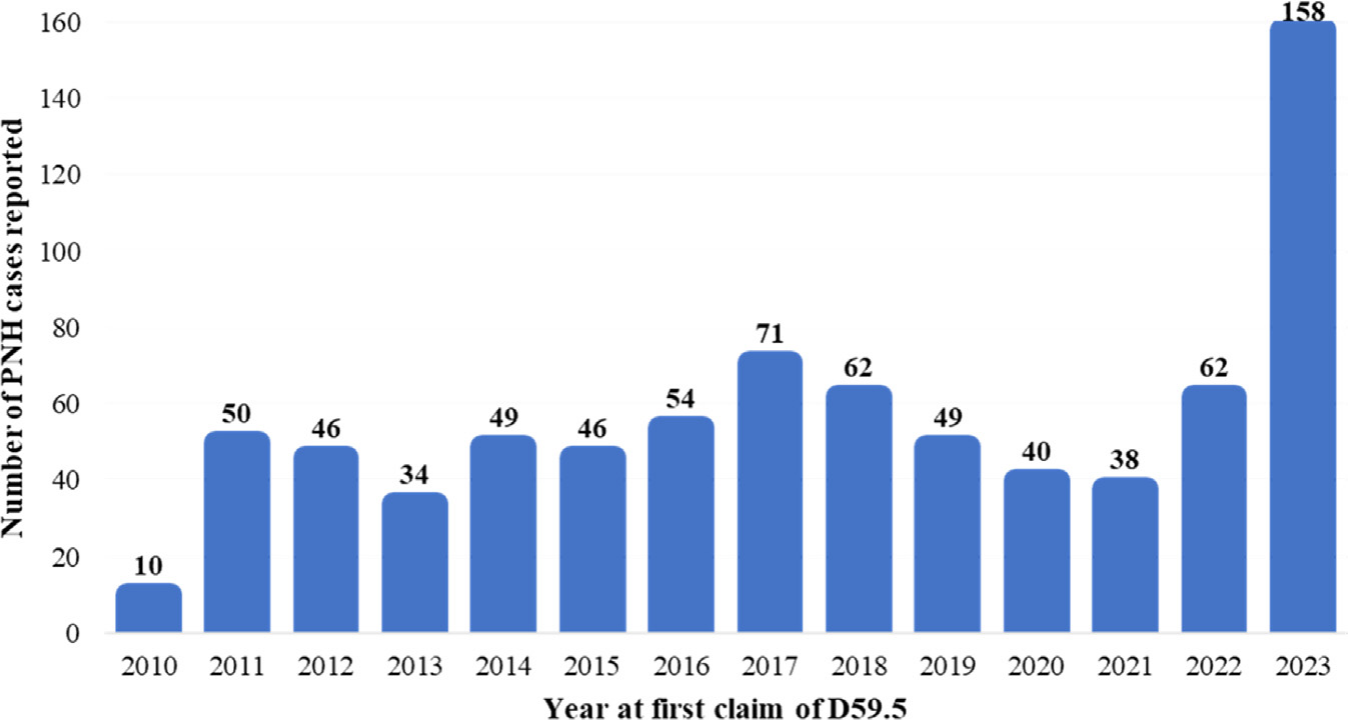
Paroxysmal nocturnal hemoglobinuria cases reported for each year in the study period.

A total of 769 individuals with at least one claim of ICD-10 D59.5 were identified in the database from 2010-2023. The number of PNH cases reported for each year of the study period is summarized in Figure 2.

The year with the most PNH diagnostic records was 2023 with 158 (20.5%) cases.

Of the identified PNH patients (n = 769), the median age at index date was 41.0 (interquartile range [IQR]: 27.0) years old. There was a slight predominance of females (50.3%). Most of the population was White (50.1%) and lived in the southeastern region of Brazil (39.0%). The mean follow-up time for patients was 1.1 (standard deviation [SD]: 2.0) years. Demographic characteristics are summarized in Table 1.

**Table 1:** Demographic characteristics of paroxysmal nocturnal hemoglobinuria patients.

Characteristic	Total	Treatment	
		Eculizumab (at any time)	Without eculizumab
n (%)	**769 (100)**	**326 (42.4)**	**443 (57.6)**
**Age at first claim (index date) - years**			
Valid - n (%)	**769 (100)**	**326 (42.4)**	**443 (57.6)**
Median (min - max)	41.0 (1.0 - 92.0)	43.0 (11.0 - 92.0)	38.0 (1.0 - 92.0)
IQR (Q3-Q1)	27.0 (56.0 - 29.0)	25.0 (57.0 - 32.0)	28.0 (55.0 - 27.0)
**Gender** – n (%)			
valid	**769 (100)**	**326 (42.4)**	**443 (57.6)**
Female	387 (50.3)	171 (52.5)	216 (48.8)
Male	382 (49.7)	155 (47.5)	227 (51.2)
**Race** - n (%)			
valid	**685 (100)**	**325 (47.4)**	**360 (52.6)**
White	343 (50.1)	162 (49.8)	181 (50.3)
Brown	44 (6.4)	23 (7.1)	21 (5.8)
Black	274 (40.0)	132 (40.6)	142 (39.4)
Yellow	24 (3.5)	8 (2.5)	16 (4.4)
**Region of residence** - n (%)			
valid	**769 (100)**	**326 (42.4)**	**443 (57.6)**
Southeast	300 (39.0)	93 (28.5)	207 (46.7)
Northeast	203 (26.4)	110 (33.7)	93 (21.0)
South	145 (18.9)	70 (21.5)	75 (16.9)
Midwest	73 (9.5)	34 (10.4)	39 (8.8)
North	48 (6.2)	19 (5.8)	29 (6.5)
Follow-up time*, years			
valid n (%)	**769 (100)**	**326 (42.4)**	**443 (57.6)**
Mean (SD)	1.1 (2.0)	0.7 (1.4)	1.3 (2.2)
Median (min - max)	0.3 (0.0 - 12.3)	0.3 (0.0 - 10.2)	0.2 (0.0 - 12.3)
IQR (Q3 – Q1)	0.8 (0.8 - 0.0)	0.4 (0.5 - 0.1)	1.6 (1.6 - 0.0)

SD: standard deviation; Min: minimum value; Max: maximum value; IQR: interquartile range.

⁎ Time since index date (ICD-10) up to the last date of available patient information in the database.

A total of 326 (42.4%) PNH patients underwent treatment with eculizumab. Patients had a median age of 43.0 (IQR: 25.0) years old at the index date. The majority were women (52.5%) and nearly half identified themselves as White (49.8%), with the predominant region of residence being the Northeast (33.7%) of Brazil. The average follow-up time for patients who received eculizumab was 0.7 (SD: 1.4) years (Table 1).

### Patient treatment

The mean time from the index date to the start of PNH treatment was 191.6 (SD: 389.4) days. For patients treated with eculizumab, the mean time was 172.9 (SD: 372.5) days. The mean PNH treatment duration of the entire cohort was 554.0 (SD: 900.7) days. Of the patients treated with eculizumab, the mean treatment duration was 628.8 (SD: 999.5) days (Table 2).

**Table 2:** Characteristics of patient’s treatment.

	Total	Treatment	
		Eculizumab (at any time)	Without eculizumab
**Patients** - n (%)	**769 (100)**	**326 (42.4)**	**443 (57.6)**
**Time from the index date until PNH treatment (days)**			
valid - n	**369**	**236**	**133**
Mean (SD)	191.6 (389.4)	172.9 (372.5)	263.6 (436.7)
**Duration of PNH treatment (days)**			
valid - n	**369**	**236**	**133**
Mean (SD)	554.0 (900.7)	628.8 (999.5)*	464.9 (504.1)

SD: standard deviation.

⁎ Treatment duration reflects the span between first and last eculizumab infusion, which may not align with follow-up time.

### Distance analysis

Of the 769 PNH patients, the distance from the patient’s residence to specialized medical units was measured for 677 (88.0%) patients. For patients not receiving eculizumab (n = 363), the distance was calculated based on the specialized medical unit they visited most frequently. For patients treated with eculizumab (n = 314), the location was defined by the requesting establishment for 38 (12.1%) patients and by the most frequent specialized medical unit for the remaining 276 (87.9%).

The spatial analysis revealed significant variability in the distance analysis, with a mean distance of 82.5 (SD: 225.0) km. Patients residing in the northern region had to travel an average of 149.1 (SD: 361.4) km. Those living in the southern, midwestern, northeastern, and southeastern regions traveled 116.4 (357.2) km, 83.0 (113.3) km, 73.2 (180.8) km, and 62.7 (151.2), respectively (Table 3).

**Table 3:** Evaluation of the distances from the patient’s residence to specialized medical units according to eculizumab treatment.

	Total	Treatment	
		Eculizumab at any time	Without eculizumab
**Total cases**			
Valid – n (%)	677 (100)	314 (46.4)	363 (53.6)
Distance (km) - Mean (SD)	82.5 (225.0)	87.5 (265.2)	78.2 (183.0)
**Region of residence: North**			
Valid – n	42	18	24
Distance (km) - Mean (SD)	149.1 (361.4)	176.4 (253.8)	125.8 (438.3)
**Region of residence: Northeast**			
Valid – n	181	102	79
Distance (km) - Mean (SD)	73.2 (180.8)	64.5 (216.8)	84.4 (119.6)
**Region of residence: Midwest**			
Valid – n	65	35	30
Distance (km) - Mean (SD)	83.0 (113.3)	61.7 (82.2)	107.8 (138.6)
**Region of residence: South**			
Valid – n	127	69	58
Distance (km) - Mean (SD)	116.4 (357.2)	156.8 (468.4)	67.4 (117.7)
**Region of residence: Southeast**			
Valid – n	262	90	172
Distance (km) - Mean (SD)	62.7 (151.2)	52.6 (63.4)	68.0 (180.9)

SD: standard deviation; km: kilometer.

Among patients treated with eculizumab (at any time), the average distance traveled was 87.5 (265.2) km. The average distance varied from 52.6 (63.4) km in the southeastern region, 61.7 (82.2) in the Midwest, 64.5 km (216.8 km) in the Northeast, 156.8 km (468.4 km) in the South, to 176.4 (253.8) km in the northern region of the country (Table 3).

### Eculizumab persistence analysis

Among eculizumab users (n = 326), in the patient follow-up approach, 257 (78.8%) PNH patients were classified as ‘persistent’ and 69 (21.2%) as ‘non-persistent.’ Considering the interval between the first and last eculizumab records, most patients (n = 199; 61.0%) were classified as ‘non-persistent’ (Table 4).

**Table 4:** Eculizumab persistence analysis.

Approach	Eculizumab users (n = 326)	
	Persistent	Non-persistent
Patient follow-up	257 (78.8%)	69 (21.2%)
Interval between first and last eculizumab records	127 (39.0%)	199 (61.0%)

### Focus group: Results of the paroxysmal nocturnal hemoglobinuria patient experience

Patients in the focus group reported being unaware of PNH before being diagnosed, often receiving a diagnosis after presenting with complications. Although some patients are diagnosed after the first symptoms, some cases can take months between the first symptoms and a correct diagnosis.

**Patient 2:***"I was surprised when I found out I had it, and I hadn't sought medical help for PNH. I sought help because I had a liver issue and had a stroke. I had to undergo a liver transplant and was left with this sequel from the stroke. [...] I underwent the transplant, only to then discover the disease."*

**Patient 5:***"I had the first symptoms, but I was diagnosed with bone marrow aplasia. From there, I had relapses, I had a stroke. In 2005, I underwent treatment with thymoglobulin. […] it was then that the hematologist and researcher at the clinic said, 'let's do a test just to clear my doubt.' When he did in 2008, it turned out to be PNH."*

Treatment with eculizumab significantly affects daily activities and professional life. Patients reported losing a significant part of the day to treatment, especially when they had to undergo treatment every two weeks, impacting both employment and quality of life.

**Patient 4:***"In my case, it affected me a lot. I am unemployed, but it did impact me because I did the afternoon [treatment] and ended up losing the whole afternoon. And there was also a time when I did the medication pickup, which was on another day."*

The distance to the treatment center varies among patients. Transportation is one of the difficulties faced, with many patients reporting using public transport, which can be slow and tiring.

**Patient 1**: *"Generally, I go by bus, but sometimes I also go with my daughter."*

One of the main difficulties mentioned is the time spent at the treatment center, with long periods of waiting to receive the infusion. Additionally, the need to leave home very early for treatment sessions was highlighted as a constant challenge.

**Patient 3:***"The travel time, we know patients who practically leave the night before to get treatment and arrive the next morning, stay all day, then sometimes return the day after. As it's treatment in a public hospital, sometimes we wait a long time there, so imagine a person who works, who needs to be present at work and whose manager won't understand all this every 2 weeks. The infusion time, the travel, and the time we spend at the hospital unit, are the challenges."*

**Patient 5:***"Besides the time you spend. I think today we are very repetitive. The tests, the paperwork to get the medication exemption and then there’s a year I have to pay to do it. There's a disease that has no cure, but every six months I have to be there paying and doing new tests to prove that I have it. For me, I think that’s harder."*

The responses of the patients regarding the impact of PNH on their lives ranged from fear to hope. Many expressed hopes for new medications that could simplify treatment, reducing the need for frequent infusions. Others mentioned gratitude for being alive and expecting improvements in available treatments.

**Patient 5:***"New medications emerging to stop infusing every 15 days"*

**Patient 6:***"I really believe new medications will come, that a lot of genetic therapy projects are being studied. We might be alive to see it or not, but I hope there is a cure waiting for us in the future."*

## Discussion

The findings of this study highlight significant disparities in access to treatment for PNH in SUS. Analysis revealed that patients travel, on average, 82 km to receive eculizumab treatment with notable regional variations. For example, patients in the northern region traveled an average of 149 km, compared to 62 km in the Southeast. These findings reflect geographic inequities in access to specialized care and may contribute to treatment discontinuation, particularly in underserved regions. These results are consistent with previous studies that have shown that geographic barriers can significantly impact access to high-complexity treatments in Brazil [14].

This study identified 769 individuals with at least one claim of ICD-10 D59.5 between 2010 and 2023. Most PNH patients (n = 158; 20.5%) were reported in 2023, which may be attributed to the availability of eculizumab in SUS from September 2022 [8]. This policy change may have led to increased awareness among healthcare providers, improved diagnostic efforts, and more frequent coding of PNH in administrative databases. Additionally, the availability of a high-cost treatment like eculizumab may have encouraged more accurate documentation and reporting of PNH cases to ensure access to therapy.

The spatial analysis demonstrated that patients residing in regions with lower Human Development Index (HDI) scores were required to travel significantly longer distances to access specialized treatment centers. This pattern aligns with prior studies highlighting regional disparities in healthcare accessibility across Brazil [15]. Beyond geographic challenges, patients also reported additional barriers such as inadequate transportation infrastructure, financial hardship, and delays in receiving medical attention. Collectively, these factors may contribute to reduced adherence and the high rates of non-persistence observed among eculizumab-treated patients.

Among eculizumab users, 69 (21.2%) and 199 (61.0%) of PNH patients were classified as ‘non-persistent’ according to the first and second approaches of persistence analysis, respectively. These findings reflect a substantial burden of treatment discontinuation and are consistent with prior research indicating that the economic burden of treatment, including direct medical costs and productivity loss, can negatively impact adherence [16]. Furthermore, previous studies have identified infusion frequency as a critical factor influencing both treatment preference and long-term persistence [17,18].

The qualitative findings from the focus group provided additional context to the quantitative data. Patients reported that treatment with eculizumab disrupted their daily routines and professional responsibilities due to long travel times, waiting periods, and the frequency of infusions. These insights are consistent with previous literature on the burden of anti-C5 therapies, which require hospital-based administration and are associated with reduced quality of life and work productivity [19,20]. However, it is important to note that the focus group findings are descriptive and based on a small, self-selected sample. Therefore, while they offer valuable perspectives, the results may be subject to selection bias.

The study should be interpreted in the light of several limitations. First, the analysis was restricted to patients who had at least one recorded procedure or hospitalization in the DATASUS databases. As a result, individuals with PNH who did not access care through the public system or who had no recorded procedures were excluded, potentially underestimating the total number of patients with the disease.

Second, the retrospective design and reliance on administrative claims data introduce inherent limitations, including the possibility of incomplete records, misclassification, and information bias. These challenges are common in studies using secondary data sources and have been documented in similar research [21,22].

Another methodological limitation relates to the calculation of travel distances. Due to differences in data availability, the reference point for distance estimation varied between patient groups. For eculizumab-treated patients, the requesting establishment was prioritized; for others, the most frequently visited specialized unit was used. When these units were pharmacies or regional health divisions, which do not provide infusions, the postal code of the nearest public hospital was used as a proxy. Although standardized rules were applied to minimize bias, this approach may not fully capture the actual care location, and caution is warranted when interpreting geographic comparisons.

Additionally, persistence analysis was limited to patients treated with eculizumab, as this group had structured and consistent infusion data. Non-eculizumab therapies lacked standardized documentation, precluding reliable persistence measurement. While this focus ensures methodological rigor, it may limit the generalizability of findings to the broader PNH population.

The static nature of the dataset also presents a limitation. Although DATASUS is regularly updated by the Brazilian Ministry of Health, the dataset used in this study represents a static snapshot extracted at a specific point in time and was not updated thereafter. As such, it does not reflect subsequent updates. This may result in underreporting of recent procedures, particularly for patients whose treatment occurred near the end of the observation window, potentially leading to misclassification of persistence status.

Furthermore, the lack of clinical data in the DATASUS database precluded assessment of treatment response, adverse events, or reasons for discontinuation. As such, we could not determine whether non-persistence was due to clinical factors or logistical barriers. Future studies incorporating clinical outcomes are needed to better understand these dynamics.

Finally, although ravulizumab was approved by ANVISA in 2019, it was only incorporated into SUS in 2024. As the focus group was conducted prior to or during its early implementation, none of the participants had transitioned to ravulizumab. This limits the study’s ability to assess the potential impact of newer therapies on treatment burden and adherence.

## Conclusions

The findings of this study provide a comprehensive overview of the profile of PNH patients in SUS, as well as how variability in distance may highlight potential disparities in access to health care. Non-persistence with eculizumab treatment may be associated with a higher patient burden, potentially causing difficulties in achieving better treatment adherence. The availability of alternative therapies for PNH that could facilitate patient access and ensure equitable healthcare delivery for PNH patients may help overcome the differences.

## Authors’ contributions

RDC and PMC contributed equally to the development of the manuscript and share first authorship. AB designed the study. ABMA and LPL developed the first draft of the manuscript. LVC was responsible for the linkage and quantitative analysis. NM and FF were responsible for the focal groups. All authors contributed equally to the writing of the final version of the manuscript.

## Data availability statement

The data that support the findings of this study are available from the corresponding author upon reasonable request.

## Conflicts of interest

Alessandro Bigoni, Ana Beatriz Machado de Almeida, Erica Ferreira, Diego Kashiura, and Felipe Thies are Novartis’ employees. Lays Paulino Leonel, and Lucas Vieira Cortez are IQVIA Solutions, Brazil employees. Nina Melo and Fabio Fedozzi are employees of the Brazilian Lymphoma and Leukemia Association, Paula de Melo Campos is an employee of the Universidade Estadual de Campinas, and Rodolfo Delfini Cançado is an employee of the Faculdade de Ciências Médicas da Santa Casa de São Paulo.
